# Pro Nerve Growth Factor and Its Receptor p75NTR Activate Inflammatory Responses in Synovial Fibroblasts: A Novel Targetable Mechanism in Arthritis

**DOI:** 10.3389/fimmu.2022.818630

**Published:** 2022-03-04

**Authors:** Luciapia Farina, Gaetana Minnone, Stefano Alivernini, Ivan Caiello, Lucy MacDonald, Marzia Soligo, Luigi Manni, Barbara Tolusso, Simona Coppola, Erika Zara, Libenzio Adrian Conti, Angela Aquilani, Silvia Magni-Manzoni, Mariola Kurowska-Stolarska, Elisa Gremese, Fabrizio De Benedetti, Luisa Bracci-Laudiero

**Affiliations:** ^1^ Department of Immunology, Laboratory of ImmunoRheumatology, Bambino Gesù Children’s Hospital, Istituto di Ricovero e Cura a Carattere Scientifico (IRCCS), Rome, Italy; ^2^ Division of Rheumatology, Fondazione Policlinico Universitario A. Gemelli Istituto di Ricovero e Cura a Carattere Scientifico (IRCCS), Università Cattolica del Sacro Cuore, Facoltà di Medicina e Chirurgia, Rome, Italy; ^3^ Inflammatory Arthritis Centre Versus Arthritis (RACE), Institute of Infection, Immunity and Inflammation, University of Glasgow, Glasgow, United Kingdom; ^4^ Institute of Translational Pharmacology (IFT-CNR), Consiglio Nazionale delle Ricerche, Rome, Italy; ^5^ National Centre for Rare Diseases, Istituto Superiore di Sanita’, Rome, Italy; ^6^ Confocal Microscopy Core Facility, Research Center, Bambino Gesù Children’s Hospital, Istituto di Ricovero e Cura a Carattere Scientifico (IRCCS), Rome, Italy; ^7^ Division of Rheumatology, Bambino Gesù Children’s Hospital, Istituto di Ricovero e Cura a Carattere Scientifico (IRCCS), Rome, Italy

**Keywords:** synoviocytes, inflammation, p75NTR inhibition, arthritis, nerve growth factor

## Abstract

We have recently provided new evidence for a role of p75NTR receptor and its preferential ligand proNGF in amplifying inflammatory responses in synovial mononuclear cells of chronic arthritis patients. In the present study, to better investigate how activation of the p75NTR/proNGF axis impacts synovial inflammation, we have studied the effects of proNGF on fibroblast-like synoviocytes (FLS), which play a central role in modulating local immune responses and in activating pro-inflammatory pathways. Using single cell RNA sequencing in synovial tissues from active and treatment-naïve rheumatoid arthritis (RA) patients, we demonstrated that p75NTR and sortilin, which form a high affinity receptor complex for proNGF, are highly expressed in PRG4^pos^ lining and THY1^pos^COL1A1^pos^ sublining fibroblast clusters in RA synovia but decreased in RA patients in sustained clinical remission. In *ex vivo* experiments we found that FLS from rheumatoid arthritis patients (RA-FLS) retained *in vitro* a markedly higher expression of p75NTR and sortilin than FLS from osteoarthritis patients (OA-FLS). Inflammatory stimuli further up-regulated p75NTR expression and induced endogenous production of proNGF in RA-FLS, leading to an autocrine activation of the proNGF/p75NTR pathway that results in an increased release of pro-inflammatory cytokines. Our data on the inhibition of p75NTR receptor, which reduced the release of IL-1β, IL-6 and TNF-α, further confirmed the key role of p75NTR activation in regulating inflammatory cytokine production. In a set of *ex vivo* experiments, we used RA-FLS and cultured them in the presence of synovial fluids obtained from arthritis patients that, as we demonstrated, are characterized by a high concentration of proNGF. Our data show that the high levels of proNGF present in inflamed synovial fluids induced pro-inflammatory cytokine production by RA-FLS. The blocking of NGF binding to p75NTR using specific inhibitors led instead to the disruption of this pro-inflammatory loop, reducing activation of the p38 and JNK intracellular pathways and decreasing inflammatory cytokine production. Overall, our data demonstrate that an active proNGF/p75NTR axis promotes pro-inflammatory responses in synovial fibroblasts, thereby contributing to chronic synovial inflammation, and point to the possible use of p75NTR inhibitors as a novel therapeutic approach in chronic arthritis.

## Highlights

Many chronic arthritis patients do not reach clinical remission (up to 50% for adult rheumatoid arthritis and 30% in juvenile idiopathic arthritis) with the available therapies. Identification of novel inflammatory mechanisms and biomarkers will help to identify new strategies for the treatment of arthritis patients. Our study focused on NGF and its receptor system. As a number of studies demonstrated, NGF directly modulates functions of immune cells and is involved in the regulation of the inflammatory response. Our working hypothesis was that the activation of NGF receptor pathways could play a role in the pathogenesis of arthritis. Our results demonstrated for the first time the involvement of proNGF, the immature form of NGF, and its specific receptor p75NTR, in activating pro-inflammatory responses in synovial fibroblasts, which play a central role in modulating joint inflammation. Our study demonstrated that inhibition of p75NTR/proNGF axis inhibits inflammatory response in rheumatoid arthritis synovial fibroblasts by reducing the production of cytokines that promote inflammation. These findings suggest that neutralization of p75NTR may represent a novel targetable pathway in chronic arthritis.

## Introduction

The basal production of nerve growth factor (NGF), that regulates peripheral innervation of tissues and organs, is enhanced during inflammatory responses in epithelial, muscular and endothelial tissues (1). Inflammatory cytokines regulate NGF production in several cell types (1) including fibroblasts (2, 3) and, not surprisingly, a number of studies have shown that NGF levels correlate with the magnitude of the inflammatory response in several chronic inflammatory diseases (1). High levels of NGF are reported in serum and synovium of patients with chronic arthritis (4–6) as well as in animal models of the disease (7, 8).

At the time these studies on inflammatory diseases were performed it was not possible to discriminate between the relative concentrations of mature NGF and the immature proNGF forms.

NGF is synthesized as a precursor, proNGF (9), which is processed into the mature form either in the Golgi or in the extracellular space (9, 10). Studies in neurons have shown that the immature proNGF form is not an inactive precursor (11) but binds with high affinity to the p75NTR/sortilin receptor complex (12) inducing effects that differ from those induced by mature NGF, which binds preferentially to TrkA. ProNGF activates apopototic mechanism in neuronal cells while mature NGF, through TrkA, regulates neuronal survival and phenotype maintenance (11, 13, 14). *In vivo* studies on neurodegenerative diseases and on diabetes suggest that proNGF is even more abundant than mature NGF in brain and peripheral tissues (15–17). At present, very little is known about the relative concentrations of proNGF and mature NGF in inflammatory diseases and the specific effects of proNGF and mature NGF on inflammatory responses are not yet clear.

Previous data in animal models of inflammatory diseases such as experimental autoimmune encephalomyelitis and inflammatory bowel disease (18, 19), suggested that the administration of mature NGF induces an improvement in symptoms, without providing any mechanistic information. Using Toll-like receptor 2 and 4 ligands to activate monocytes, we have demonstrated that mature NGF, through binding to TrkA, reduces the production of pro-inflammatory cytokines and increases the production of the anti-inflammatory cytokines, IL-1RA and IL-10, activating an Akt dependent intracellular signaling pathway (20). This regulatory mechanism seems defective in patients with chronic arthritis: they are characterized by a marked decrease in TrkA expression in peripheral blood and synovial fluid mononuclear cells (MNC), resulting in a loss of the inhibitory effect of NGF on inflammatory cytokine release that it is instead observed in healthy donor MNC (20). Subsequent studies demonstrated that blood MNC of juvenile idiopathic arthritis (JIA) patients are characterized by a marked over-expression of p75NTR and sortilin, the proNGF high affinity receptor complex and a significant decrease of TrkA expression, while healthy donor blood MNC show a high TrkA and a low p75NTR expression (21). The altered p75NTR and TrkA ratio found in blood JIA MNC is even more evident in the MNC obtained from JIA synovial fluids. In inflamed synovial fluids the expression of p75NTR in the MNC is enhanced compared to the one of JIA blood MNC and correlates with clinical parameters: the more inflamed was the patient the highest was the expression of p75NTR in synovial fluid MNC (21). This change in p75NTR and TrkA ratio in arthritis patient MNC, characterized by high p75NTR and low TrkA expression, results in an increased binding affinity for the proNGF form. In *ex vivo* experiment we demonstrated that administration of proNGF increases the release of inflammatory cytokines in synovial MNC from JIA patients (21). Since proNGF concentration is extremely high in the synovial fluids (SF) of inflamed synoviae (21), we hypothesized that activation of the p75NTR/proNGF axis may play an important role in synovial inflammation.

To better characterize how an active proNGF-p75NTR pathway can regulate inflammatory response in the synovia of arthritis patients we used single cell analysis to identify the p75NTR+ cell populations in the inflamed tissue and focused on fibroblast-like synoviocytes (FLS). FLS are considered major players in the pathogenesis of chronic arthritis producing cytokines that perpetuate inflammation and proteases that contribute to cartilage destruction (22).

Our data show that specific subsets of sublining FLS from RA patients overexpress p75NTR and actively express NGF. We also demonstrated in *ex vivo* experiments that inflammatory cytokines further enhanced both the expression of p75NTR and the release of proNGF in RA-FLS, creating a pro-inflammatory loop that sustains the inflammatory response. Inhibition of p75NTR activity significantly down-regulates pro-inflammatory cytokine production indicating p75NGF inhibition as a novel target for chronic arthritis treatment.

## Materials and Methods

### Patient Samples and Cell Cultures

As a source of inflamed synovial fluids, synovial fluid (SF) was obtained from patients with juvenile idiopathic arthritis (JIA) followed at the Division of Rheumathology of Bambino Gesú Children’s Hospital. The study was approved by the local ethical committee (ID#2333_OPBG_2020) and written consent was obtained from parents of children, as appropriate. Synovial tissues (total of 8) were obtained from treatment-naïve (n=4) and in sustained clinical and ultrasound remission (n=4) RA patients at the SYNGem Biopsy Unit of the Fondazione Policlinico Universitario A. Gemelli IRCCS, as approved by the Institutional Ethics Committee (ID#6334/15). The patients involved provided signed informed consent. Diagnosis of JIA and RA were based on the International League of Associations for Rheumatology classification criteria and on 2010 EULAR/ACR criteria (23). JIA patients (n= 18) had oligoarticular, extended oligoarticular, or polyarticular JIA. Ten were females. The median age at disease at sampling was 9.4 years (IQR 2.1–12.7) and the median disease duration at sampling was 2.8 years (IQR 1.9–8.7). All patients had active disease with overt arthritis at sampling. 11 patients were untreated, 7 were receiving traditional DMARDs. Patients receiving glucocorticoids were not included.

FLS from rheumatoid arthritis patients (RA-FLS) (n=8) and osteoarthritis patients (OA-FLS) (n=6) were purchased from CliniSciences (Abbiotec, Escondido, USA). Control skin fibroblasts (CTRL) were from American Type Culture Collection (ATCC). Cells were cultured in 10% fetal bovine serum (FBS) high-glucose Dulbecco’s Modified Eagle Medium (complete DMEM) with or without the addition of different doses of human recombinant IL1-β or TNF-α (all from R&D systems, Minneapolis, MN), or LPS (Sigma Aldrich, St Louis, MO). For proNGF experiments, 200 ng/ml of a cleavage-resistant proNGF that has an R-to-G substitution at amino acid position 104 (Alomone Labs, Jerusalem, Israel) were used to stimulate cells cultured in AIMV Serum Free Medium (Life Technologies, Rockville, Maryland, USA). Supernatants were collected after 18 hours of incubation before IL-6 production reaches a plateau (24). For p75NTR inhibition, cells were pre-treated for 1 hour with LM11A-31 (25) (a kind gift of Dr. Frank Longo, Stanford University) or with anti-p75NTR antibodies (clone ME20.4 Millipore, Billerica, MA, USA). *Ex vivo* experiments were performed with RA-FLS cultured in complete DMEM with or without the addition of 30% v/v synovial fluid. Cells were pre-treated 1 hour at 37°C with or without LM11A-31 or the anti-IL-1β monoclonal antibody canakinumab (5 μg/ml). (26)

### Single Cell-RNA Sequencing of Synovial Tissue

Methods for single-cell RNA sequencing (scRNAseq) of synovial tissue have already been described in detail (27). Briefly, synovial tissue biopsies were collected from naive to treatment RA patients (n=4) and from RA patients in sustained clinical and ultrasound remission (n=4) at the SYNGem Biopsy Unit of the Fondazione Policlinico Universitario A. Gemelli IRCCS using US-guided minimally invasive technique (28). High-quality total RNAs (RIN >8) were used to construct Illumina mRNA sequencing libraries. cDNA synthesis and amplification were performed by using SMART-seq v4 Ultra Low Input RNA Kit for Sequencing (cat. no. 634890, Takara) starting with 10 ng of total RNA, following the manufacturers protocol. 10 ng of amplified cDNAs were sheared prior to preparing the final libraries using the Bioruptor^®^ Pico system (Diagenode, 24 cycles of 30 sec on and 30 sec off). Dual indexed Illumina sequencing libraries were prepared by SMARTer^®^ ThruPLEX^®^ DNA-seq 48D Kit (cat. no. R400406, Takara) following the kit protocol. The pooled libraries were sequenced at Glasgow Polyomics (Glasgow, UK) on a NovaSeq 6000 system using a read length of 100 bases in paired-end mode. The reads were mapped with STAR (version 020201) with default parameter against the Human genome version GRCh38, release 91. The read count matrix was constructed with featureCounts (Version 1.6.4) using default parameters. All differential expression analysis was performed in R using the DESeq2 package. All genes with an adjusted p value < 0.05 and a log fold change of > +/- 1,5 were considered significantly differentially expressed. All raw and processed data were deposited at EMBL-EBI and are available with the accession number E-MTAB-8322.

### RNA Extraction and Real-Time PCR Analysis

After RNA extraction using Trizol Reagent (Thermo Fisher Scientific, MA), cDNAs were retro*-*transcribed using the Superscript Vilo kit (Invitrogen, CA). Real-time PCRs were performed using TaqMan Universal PCR Master Mix and gene expression assays from Applied Biosystems (CA, USA). TrkA, p75NTR, NGF, sortilin mRNA expressions were tested using Assays on Demand reagents (TrkA Hs01021011_m1; p75NTR Hs00182120_m1; NGF Hs00171458_m1; sortilin Hs00361760_m1). TaqMan Endogenous Control human HPRT Hs 02800695_m1 and GAPDH Hs 99999905_m1 (Applied Biosystems) were used as housekeeping genes. Normalized gene expression levels were calculated as

2^-ΔCt^ [ΔCt = Ct (gene of interest) – Ct (housekeeping gene)] and results were expressed in arbitrary units (A.U.). Fold changes were calculated using the 2^-ΔΔCt^ equation [ΔΔCt = ΔCt (treated sample) – ΔCt (untreated sample)] (29).

### Cytokine and NGF-proNGF ELISA

Levels of IL-6, MCP1, IL-8 were analyzed using R&D Quantikine ELISA. Conditioned media and SF were assayed using ELISAs specific for human mature NGF or proNGF, as described (30).

### Western Blot Analysis

After RIPA buffer lysis (Cell Signaling, Leiden, The Netherlands), protein concentration was measured with BCA Protein assay (Thermo Fisher Scientific). 30 μg protein extracts were resolved by 10% SDS PAGE, transferred to nitrocellulose membranes and probed with antibodies against: phospho-stress-activated protein kinase/c-Jun NH(2)-terminal kinase (SAPK/JNK) (Thr183/Tyr185, clone G9 MAB #9255), total SAPK/JNK (#9252), phospho-p38 Mitogen-Activated Protein Kinase (MAPK) (Thr180/Tyr182, clone 28B10 MAB #9216), total p38 MAPK (#9212) (all from Cell Signaling), p75NTR (clone 8211 MAB5264) (EMD Millipore), GAPDH (clone G9, MAB #32233) (Santa Cruz Biotechnology), and tubulin (Sigma Aldrich). Blots were developed by ECL system (Amersham Biosciences) according to the manufacturer’s protocol.

### Immunofluorescence Analysis

RA-FLS and OA-FLS were fixed in 4% paraformaldehyde, permeabilized with 0.5% Triton X-100 PBS, incubated for 1 hour at RT with 1% BSA, 5% goat serum (Abcam, Cambridge, UK) PBS and then with mouse anti-p75NTR antibody (Merck, Darmstadt, Germany, clone 8211) or rabbit anti-proNGF (Merck AB9040) and Alexa Fluor secondary antibodies (Invitrogen). Confocal imaging acquisition was performed on Olympus Fluoview FV1000 confocal microscope using a 40× (0.90 NA oil) objective.

### Apoptosis Detection

Cells were incubated with or without LM11A-31 (10, 100 nM) for 1 hour at 37°C and then 30% v/v SF was added. After 18 hours apoptotic cells were stained using Annexin V-FITC Apoptosis Detection Kit (Sigma Aldrich). Cells were analyzed by flow cytometry (FACSCanto II, BD Biosciences CA, USA).

### Statistical Analysis

Data are presented as mean ± standard error of the mean (SEM). Statistical analysis was performed using GraphPad Prism 5 Software (GraphPad Software, La Jolla, CA). Statistical significance is shown as *p<0.05 **p<0.01 and ***p<0.001.

## Results

### p75NTR Expression Is Markedly Increased in Distinct Synovial Fibroblasts Clusters From RA

**Figure 1:**
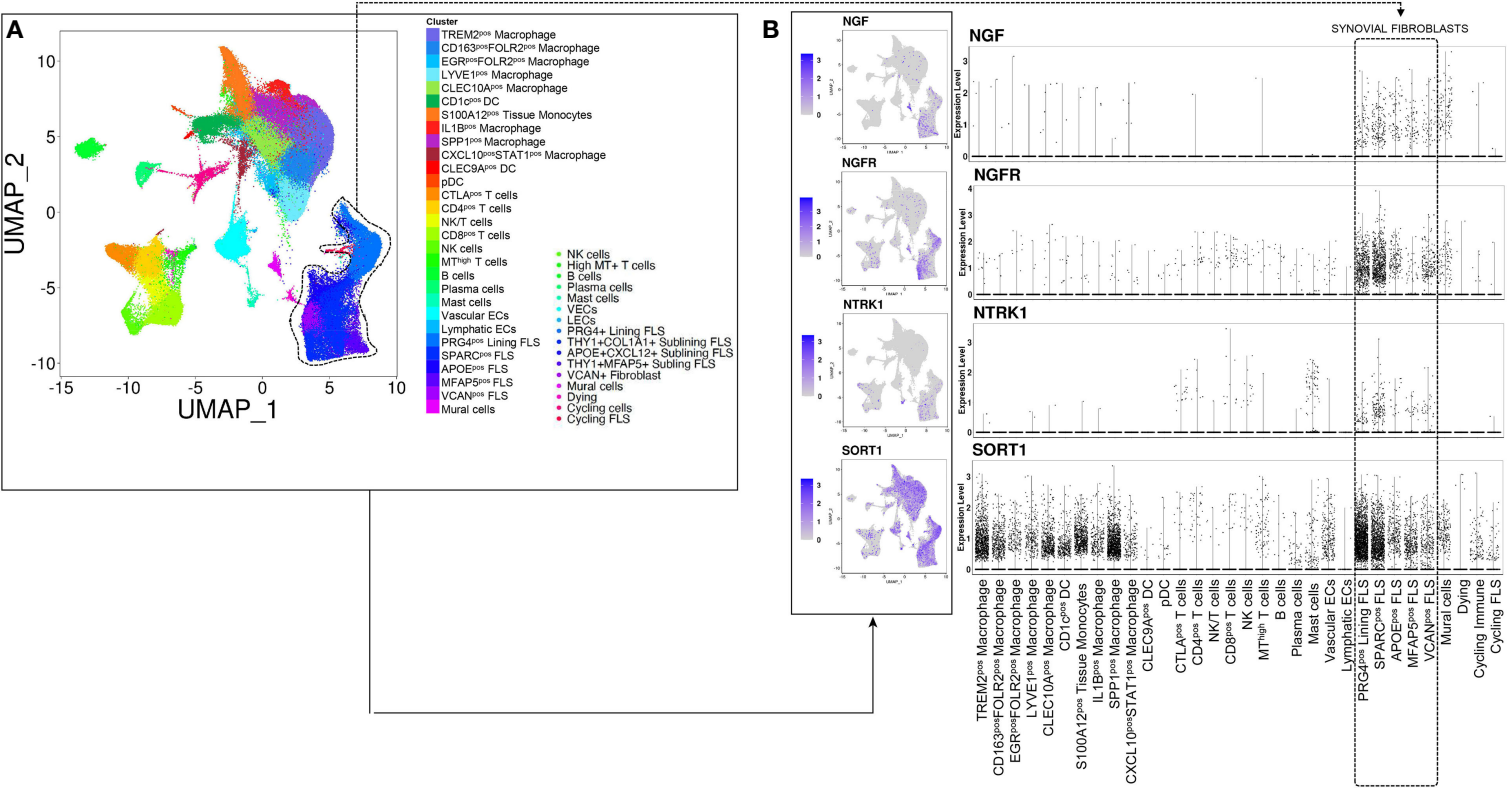
NGF, p75NTR, TRKA and SORT1 are expressed in lining and sublining synovial fibroblasts clusters from treatment-naïve patients with rheumatoid arthritis. **(A)** UMAP (Uniform Manifold Approximation and Projection for Dimension Reduction) of synovial tissue cells from treatment-naïve Rheumatoid Arthritis patients (n = 4) (27) by scRNAseq. Each dot represents a cell and distinct cell clusters are identified according to the color legend. **(B)** UMAP and dot plots showing NGF, NGFR, NTRK1 and SORT1 gene expression in distinct inflammatory and stromal cells clusters. The violet color identifies cells expressing NGF, p75NTR, TrkA and sortilin.

To determine which immune or stromal cells might be activated by the proNGF/p75NTR axis in synovial tissue, we investigated the expression of p75NTR, TrkA and SORT1 in previously published scRNAseq data sets referring to whole synovial tissues (total 118.622 cells) derived from treatment-naïve RA patients or from RA in sustained clinical and imaging remission (**Figure 1A**) (27). Among different synovial fibroblast clusters, in treatment-naïve RA synovial tissues, lining layer PRG4^pos^ cluster and sublining THY1^pos^COL1A1^pos^ fibroblast cluster are enriched of p75NTR compared to other FLS clusters as well as to other resident and inflammatory synovial cells such as macrophages or T and B lymphocytes (**Figure 1B**). Moreover, as shown in **Supplementary Figure 1**, scRNAseq analysis revealed that, when compared to treatment-naïve RA, p75NTR and TrkA expression is significantly reduced in THY1^pos^COL1A1^pos^ fibroblast cluster in RA patients in sustained clinical and imaging remission. We also observed a significant reduction of SORT1 expression in lining layer (PRG4^pos^ cluster) as well as in sublining layer fibroblasts (THY1^pos^COL1A1^pos^).

### Expression of p75NTR and proNGF Release by RA Synovial Fibroblasts

**Figure 2:**
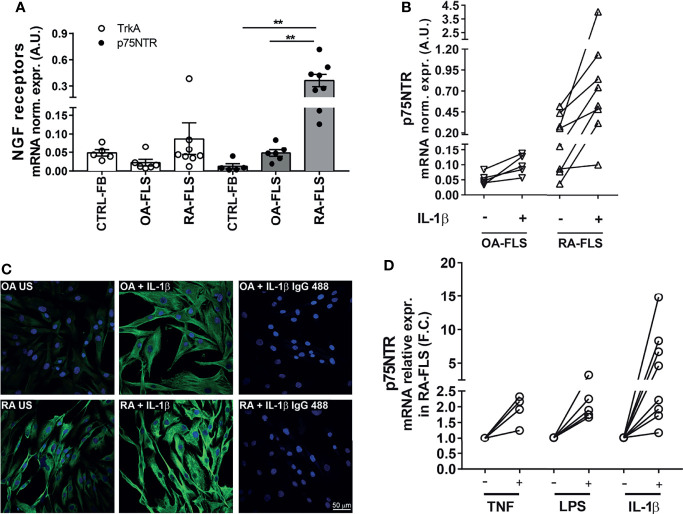
NGF receptor levels in synovial fibroblasts from RA patients. **(A)** FLS from RA patients (RA-FLS) (n = 8) have lower TrkA and higher p75NTR mRNA expression levels than OA-FLS (n = 6), and skin fibroblasts (CTRL-FB) (n = 5). Results are reported in arbitrary units (A.U.) after normalization with *HPRT* expression. Data were analyzed using unpaired *t*-test (**p < 0.01). **(B)** 1 ng/ml IL-1β up-regulates p75NTR expression in RA-FLS (n = 8) compared to OA-FLS (n = 5). Results are reported in arbitrary units (A.U.) after normalization with *HPRT* expression. **(C)** Immunofluorescence analysis confirms higher basal levels of p75NTR in RA-FLS (Untreated, RA-US) than in OA-FLS (OA US). IL-1β (10 ng/ml) stimulation further enhances p75NTR in RA-FLS. Representative images of three independent experiments are shown. p75NTR immunofluorescence is shown in green, nuclei in dark blue. Scale bar 50μm. **(D)** Inflammatory stimuli, 100 ng/ml TNF-α (n = 4), 100 ng/ml LPS (n = 5), and 1 ng/ml IL-1β (n = 8), induce p75NTR expression in RA-FLS. Results are compared to unstimulated condition and calculated as Fold Change (F.C.).

Given the high *in vivo* expression of p75NTR in pathogenic synovial fibroblast clusters in RA synovium, we investigated whether its expression is maintained in RA-FLS *in vitro* and what is the biological effect of an active p75NTR pathway. We analysed p75NTR and sortilin expression also in OA-FLS because although OA is a degenerative non-autoimmune disease, it is characterized by some inflammation of the joints. To verify the basal expression of p75NTR in non-inflamed conditions we used skin fibroblasts (skin-FB) from healthy donors. As shown in **Figure 2A** p75NTR is expressed far more in RA-FLS than in either OA-FLS or skin-FB. Control skin fibroblast express higher levels of TrkA than RA-FLS. p75NTR mRNA expression data were confirmed by Western blot (**Supplementary Figure 2A**). Sortilin showed the same expression pattern as p75NTR (**Supplementary Figure 2B**).

*In vitro* stimulation with IL-1β marginally up-regulated p75NTR mRNA expression in OA-FLS (**Figure 2B**), while induced a marked dose-dependent increase in RA-FLS (**Figure 2B** and **Supplementary Figure 2C**). Immunofluorescence confirmed a higher basal expression of p75NTR and its enhancement after stimulation with IL-1β in RA-FLS, while only a slight increase was observed in OA-FLS using a maximal dose (10ng/ml) of IL-1β (**Figure 2C**). Other pro-inflammatory stimuli, such as TNF-α and LPS, also upregulate p75NTR expression in RA-FLS (**Figure 2D**).

**Figure 3:**
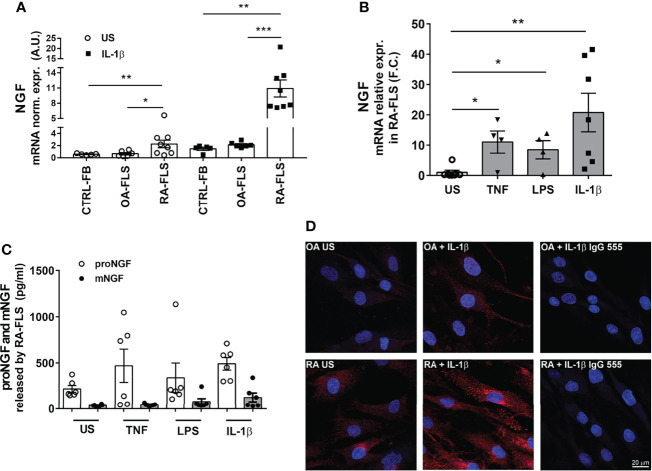
Inflammatory stimuli enhance NGF expression in synovial fibroblasts from RA patients. **(A)** Stimulation with 1 ng/ml of IL-1β induces the expression of NGF in RA-FLS (n = 8), OA-FLS (n = 6) and skin-fibroblasts (CTRL-FB) (n = 5). Results are expressed as arbitrary units (A.U.) after normalization with *HPRT.* Data were analyzed using unpaired *t*-test (**p < 0.01). **(B)** Inflammatory stimuli enhance NGF mRNA expression in RA-FLS. Basal levels of NGF expression were measured in unstimulated (US) RA-FLS (n = 7) and in RA-FLS stimulated with 100 ng/ml TNF-α (n = 4), 100 ng/ml LPS (n = 4) and 1 ng/ml IL1-β (n = 7). Results are compared to unstimulated condition and calculated as Fold Change (F.C.). Data were analyzed using unpaired *t*-test (*p < 0.05, **p < 0.01, ***p < 0.0001). **(C)** Newly developed ELISAs that discriminate between proNGF and mNGF showed that proNGF is the most abundant form in conditioned media collected from RA-FLS. proNGF production is enhanced by inflammatory stimuli (100ng/ml TNF, 100 ng/ml LPS, 1 ng/ml IL-1β) in RA FLS (n = 6). **(D)** RA FLS show an increase in proNGF basal protein synthesis after stimulation with IL-1β (1 ng/ml). A dim immunofluorescence for proNGF was observed in OA-FLS after IL-1β stimulation. Representative images of three independent experiments are shown. proNGF immunofluorescence is shown in red, nuclear staining in dark blue. Scale bar 50μm.

Compared with OA-FLS and CTRL-FB, unstimulated RA-FLS express higher basal levels of NGF mRNA. In RA FLS the already high NGF mRNA levels were further increased in a dose-dependent manner by IL-1β (**Figure 3A** and **Supplementary Figure 2D**), as well as by TNF-α or LPS (**Figure 3B**). At a protein level, proNGF is the most abundant form of NGF secreted by unstimulated RA-FLS, consistently with our previous observation that proNGF, and not mature NGF, is the predominant NGF form in synovial fluids from inflamed joints of JIA and RA patients (21). Secretion of proNGF in RA-FLS is further enhanced by IL-1β, as well as by LPS or TNF−α (**Figure 3C**). Immunofluorescence showed that proNGF is higher in unstimulated RA-FLS, than in OA-FLS, and markedly increased in the cytoplasm of RA-FLS after IL-1β-stimulation (**Figure 3D**).

### p75NTR Activation Enhances Inflammatory Cytokine Production by RA Synovial Fibroblasts

**Figure 4:**
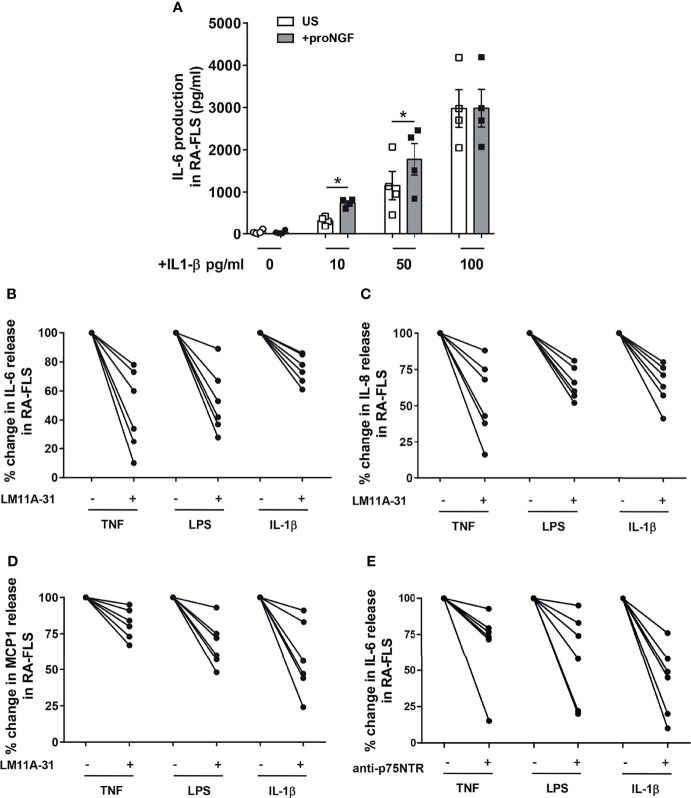
proNGF increases the expression of inflammatory cytokines in synovial fibroblasts from RA patients. **(A)** The addition of exogenous proNGF (200ng/ml) together with IL-1β at suboptimal doses (10pg/ml and 50pg/ml) induces a synergic increase in IL-6 production not observed at higher concentration of IL-1β. The experiments (n = 4) were performed in serum free medium. Data were analyzed by paired *t*-test (*p < 0.05). **(B–D)** Inhibition of proNGF binding to p75NTR with LM11A-31 (10nM) decreases inflammatory mediators: IL-6 **(B)**, IL-8 **(C)** and MCP1 **(D)** production was significantly reduced in RA-FLS cells activated using 100 ng/ml TNF-α, 100 ng/ml LPS or 1 ng/ml IL-1β. RA-FLS were cultured for 18 hours in 10% FBS DMEM. The inhibitory effect of LM11A-31 on cytokine release is expressed as percentage decrease (% decrease) from activated cells. **(E)** To obtain a confirm of the effects of p75NTR blocking, p75NTR was neutralized using a specific anti-p75NTR antibody (2,5 µg/ml) and the release of IL-6 was measured in RA-FLS activated with 100 ng/ml TNF-α, 100 ng/ml LPS or 1ng/ml IL-1β. In this set of experiments (n = 6) cells were cultured in 10% FBS DMEM for 18 hours. The results are expressed as percentage decrease (% decrease) from activated cells.

To understand the functional relevance of proNGF-p75NTR interaction, we stimulated RA-FLS with recombinant proNGF and analyzed the production of inflammatory cytokines. To avoid interference by NGF and/or proNGF present in bovine serum (31), cells were cultured in serum-free medium. The addition to RA-FLS of proNGF alone did not modify the release of IL-6 (**Figure 4A**). In contrast, the addition of proNGF together with sub-optimal concentrations of IL-1β significantly enhanced IL-6 release (**Figure 4A**). The synergy between IL-1β and proNGF was lost at higher IL-1β concentrations. Since IL-1β induces a dose-dependent increase in both p75NTR and proNGF expression in RA-FLS (**Supplementary Figures 2C, D**), the lack of effect of the exogenous proNGF addition at maximal IL-1β concentrations suggested the presence of a proNGF/p75NTR autocrine loop. Consistently with an autocrine loop, in RA-FLS stimulated with IL-1β, TNF-α or LPS without the addition of exogenous proNGF, the selective blocking of the binding site for proNGF on p75NTR, using a small non-peptide p75NTR ligand inhibitor (LM11A-31 (25), resulted in a significant decrease in inflammatory cytokine production (**Figures 4B**–**D** and **Supplementary Figures 3A**–**C**). Similarly, the blocking of p75NTR using a p75NTR-neutralizing antibody significantly decreased IL-6 production in RA-FLS stimulated with different inflammatory stimuli and without the addition of exogenous proNGF (**Figures 4E** and **Supplementary Figures 3D, E**). Moreover, when exogenous pro-NGF was added to IL-1β activated RA-FLS the LM11A-31 blocking of proNGF binding to p75NTR resulted in a more pronounced inhibition of IL-6 release than would be expected if only exogenous proNGF binding was blocked (**Supplementary Figure 4A**).

### proNGF Present in Synovial Fluid Induces Inflammatory Cytokine Production From RA-FLS

**Figure 5:**
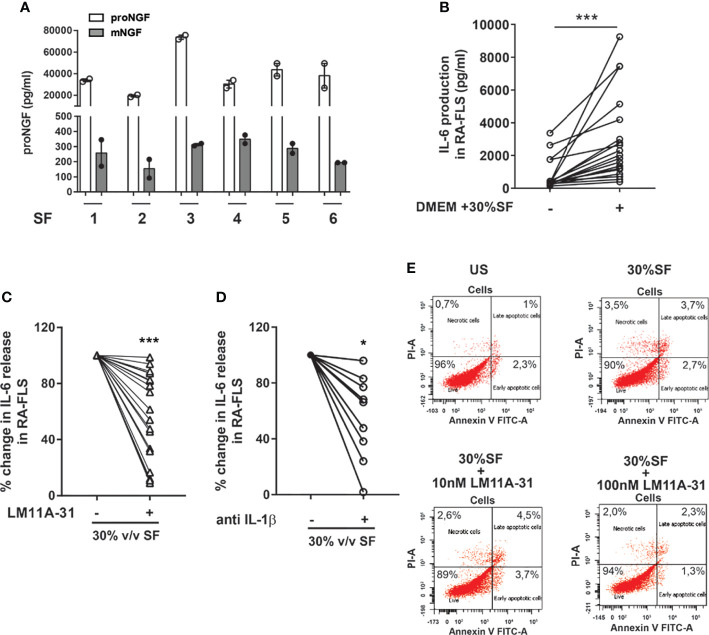
proNGF present in synovial fluids increases the expression of inflammatory mediators in synovial fibroblasts from RA patients. **(A)** proNGF is by far the most abundant NGF form (100 to 200-fold that of mature NGF) detected in synovial fluids (SF) obtained from six active patients (1-6). **(B)** To recreate *ex vivo* inflamed synovia condition, 30% v/v synovial fluid was added to RA-FLS (n = 19) cultured in 10% FBS DMEM. Data were analyzed by paired *t*-test (***p < 0.001). **(C)** RA-FLS in 30% v/v synovial fluid were treated with 10 nM of LM11A-31 with LM11A-31 and IL-6 production was measured after 18 hours. The data represent the percentage of inhibition obtained in 19 independent experiments performed using seven different RA-FLS and synovial fluids obtained from different patients (n = 18). Data were analyzed by one sample *t*-test (***p < 0.001). **(D)** 30% v/v synovial fluid was added to RA-FLS cultured in 10% FBS DMEM with or without the addition of anti-IL1β (5µg/ml) (n = 9). IL-6 release was measured after 18 hours of incubation. Data were analyzed by one sample t-test (*p < 0.05). **(E)** The apoptosis rate of RA-FLS treated with 30% v/v of synovial fluids with or without the addition of LM11A-31 at two different doses (10nM used for all our experiment and a ten-fold higher dose 100 nM) was analyzed by Annexin V/PI staining. No modification in the percentage of apoptotic cells was observed following the addition of synovial fluid or after p75NTR inhibition with LM11A-3 with both the doses used. Representative scatterplots of three independent experiments are shown.

The synovial fluids (SF) of JIA and RA patients are characterized by high concentrations of proNGF and markedly lower concentrations of mature NGF (21). To investigate the role of these high proNGF synovial concentrations, we recapitulated pathological synovial conditions *in vitro* by culturing RA-FLS in a medium supplemented with 30% v/v of SF from patients with active arthritis. As shown in **Figure 5A** and **Supplementary Figure 4B**, we further confirm that SF from arthritis patients have much higher concentration of proNGF than of mature NGF. The addition of 30% SF led to a significant increase of IL-6 release by RA-FLS (**Figure 5B**). The addition of the p75NTR inhibitor LM11A-31, by blocking the binding of the proNGF present in SF to p75NTR (highly expressed in RA-FLS), resulted in a significant inhibition (p<0.0001) of IL-6 release induced by synovial fluid stimulation (**Figure 5C**). Since it is well known that IL-1β is a potent activator of RA-FLS (32, 33), as an internal control in these experiments we neutralized the IL-1β present in the inflamed synovial fluid by using canakinumab. We found that neutralization of p75NTR using LM11A-31 was as effective as IL-1β inhibition with canakinumab (**Figure 5D**).

**Figure 6:**
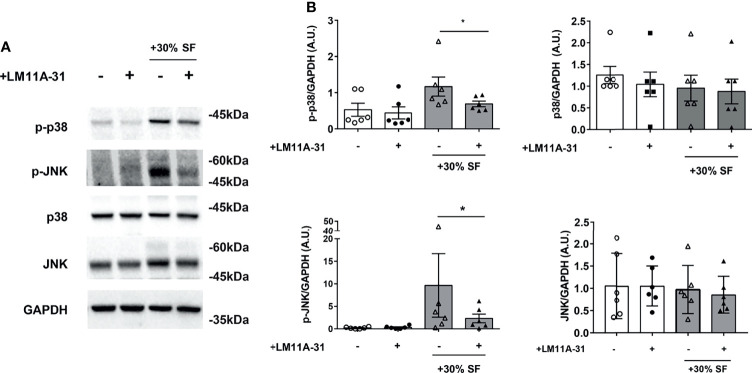
Activation of p38 and JNK is reduced by LM11A-31 inhibition of p75NTR in RA FLS **(A)** RA-FLS were starved for 3 hours and incubated with 10% FBS-DMEM supplemented with 30% v/v synovial fluid and with or without LM11A-31 (10nM) for 18 hours. A reduction in the phosphorylation of p38 and JNK was observed after LM11A-31 inhibition of p75NTR activity. *GAPDH* was used as loading control. **(B)** Densitometric analysis was normalized to the corresponding band intensity of *GAPDH*. Data were analyzed by paired *t*-test (*p < 0.05) and represent mean ± SEM of 6 independent experiments. Results are expressed as arbitrary units (A.U.).

The observed decrease in cytokine production, induced by LM11A-31 blocking of p75NTR activity, is not due to any toxic effect of the p75 inhibitor or decreased survival of RA-FLS: neither 30% v/v SF nor different LM11A-31 concentrations (the highest dose was 10-fold that used in the neutralization experiments) induced an increase in the percentage of apoptotic cells in RA-FLS (**Figure 5E**). To gain insights into the intracellular mechanisms regulated by p75NTR activation in RA-FLS, we focused on the p38 and JNK proteins, members of the MAPK pathways and key mediators of pro-inflammatory cytokine production in RA synovium (34). As shown in **Figure 6**, our data indicated that p75NTR activity modulates the pro-inflammatory signaling response. Indeed, RA-FLS cultured in 30%SF showed an increase in p38 and JNK phosphorylation. This effect is mediated by the proNGF present in inflamed SF, since p75NTR neutralization significantly reduced p38 and JNK phosphorylation. As previously observed in human FLS (35) and in an *in vivo* collagen-induced arthritis model (34), JNK was expressed only as 54 kDa isoform.

## Discussion

Despite currently available targeted therapies, many patients with RA or JIA do not achieve remission, instead accruing functional and structural damage, suggesting the imperative need of new therapeutic approaches based on novel mechanisms of action. Here, we provide the evidence for a novel pro-inflammatory role of p75NTR and its ligand proNGF in the amplification of synovial inflammatory responses.

**Figure 7:**
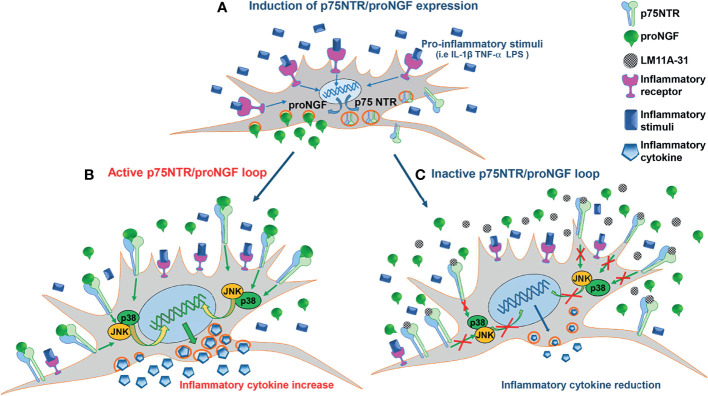
The pro-inflammatory p75NTR/proNGF loop in RA-FLS. **(A)** Inflammatory stimuli activate an autocrine loop involving proNGF and p75NTR. Inflammatory stimuli strongly induce the contemporary expression of p75NTR, the specific proNGF receptor, and of its ligand, proNGF, in RA-FLS. The increased p75NTR expression results in a higher binding capacity of RA-FLS for proNGF, whose endogenous production is strongly induced by inflammatory stimuli. **(B)** The active p75NTR/proNGF axis enhances inflammatory cytokines production. The high amounts of endogenously-produced proNGF, released by activated RA-FLS, interact with the p75NTR receptors highly expressed on RA-FLS membrane. The activation of p75NTR intracellular pathways (i.e. p38 and JNK) results in an amplified production of inflammatory cytokines (i.e. IL-6). **(C)** Inhibition of the p75NTR/proNGF loop decreases inflammatory cytokines production. The blocking of p75NTR using LM11A-31, a small molecule that specifically blocks the binding site of p75NTR for proNGF, results in a net reduction of inflammatory cytokine production (i.e. IL-6). The endogenous proNGF released by active RA-FLS cannot bind to p75NTR and, consequently, does not activate p75NTR intracellular pathways that induce inflammatory cytokine production.

We found that in RA synovium at disease onset two distinct clusters of FLS with specific tissue locations (sublining THY1^pos^COLA1^pos^ and lining PGR4^pos^ FLS respectively), that drive inflammation and damage (36), show the highest expression levels of p75NTR and of its co-receptor sortilin when compared to other synovial tissue cells. Moreover, the comparative analysis of proNGF/p75NTR axis component expression, at single cell level, on synovial tissues from treatment-naïve and remission RA patients supports the notion that proNGF/p75NTR axis is actively involved in RA synovial inflammation, being significantly repressed once sustained remission is achieved. In *in vitro* studies we confirmed that RA-FLS have a markedly higher basal expression of p75NTR and sortilin than skin fibroblasts or OA-FLS, indicating the presence of a functional high-affinity proNGF receptor complex. Inflammatory stimuli, such as LPS and the classical pro-inflammatory cytokines as IL-1β and TNF-α, enhance the expression of p75NTR in RA-FLS, as it is seen in blood and synovial fluid-derived immune cells (21), indicating a strict relationship between inflammation and the overexpression of p75NTR. FLS isolated from patients with arthritis are thus characterized by a high p75NTR and a low TrkA expression that may favor the biological effects of proNGF and dampen the anti-inflammatory actions of mature NGF mediated through TrkA activation (20). In RA-FLS, in addition to the observed overexpression of a functional p75NTR receptor complex, we found that inflammatory stimuli, among which IL-1β is the most effective, also markedly enhanced the production of large amounts of proNGF, but not of mature NGF. These data are consistent with our previous findings of high levels of proNGF, but not of mature NGF, in synovial fluids from inflamed joints of JIA and RA patients (21). Constitutively high expressions of p75NTR and enhanced release of proNGF, as well as their up-regulation induced by IL-1β, TNF-α and LPS, are specific features of RA-FLS. OA-FLS show a much lower basal expression of p75NTR and of proNGF, which are only marginally up-regulated by IL-1β. A vast body of evidence shows that OA-FLS and RA-FLS are characterized by differences in the proportion (37) of fibroblast subsets with non-overlapping functions which play distinct roles in the pathogenesis of OA and RA: in RA there is a prevalence of immune effector fibroblasts that sustain inflammation through the production of chemokines and cytokines, and of bone effector fibroblasts that mediate joint damage through the production of matrix metalloproteinases (37–40). RA-FLS have a pro-inflammatory and aggressive phenotype (41) and a number of epigenetic changes have been suggested to account for these abnormalities (42). In this contest, p75NTR overexpression and proNGF overproduction may be part of this abnormal pro-inflammatory phenotype in RA-FLS and, possibly, of the underlying epigenetic changes. It is tempting to speculate that overproduction of proNGF and overexpression of p75NTR, leading to hyperactivation of this pathway, is, at least in part, responsible for the known prolonged and exaggerated responses of RA-FLS to cytokines (43). We therefore investigated the effects of an active proNGF/p75NTR axis in RA-FLS. The addition of proNGF to sub-optimal concentrations of IL-1β synergistically up-regulated inflammatory cytokine release. At higher concentrations of IL-1β, the effects of exogenously added proNGF became negligible, possibly because IL-1β is a strong inducer of proNGF, with high amounts of proNGF being released by IL-1β-activated RA-FLS. To test the hypothesis of an active autocrine loop involving proNGF production and p75NTR expression in RA-FLS, we inhibited p75NTR using a neutralizing antibody or LM11A-31, a non-peptide ligand of p75NTR that selectively blocks the binding site of proNGF (44, 45). The blocking of the binding of the endogenously-produced proNGF to p75NTR in IL-1β-activated RA-FLS resulted in a marked decrease in inflammatory cytokine production, indicating the functional interaction of p75NTR and proNGF and the presence of a pro-inflammatory autocrine loop. Thus, RA-FLS activated by inflammatory cytokines overexpress p75NTR and, at the same time, release increased amounts of proNGF which, by binding to p75NTR, further up-regulates inflammatory cytokine production. Neutralization of p75NTR breaks this pro-inflammatory loop and significantly reduces inflammatory cytokine production as summarized in **Figure 7**.

In order to confirm these findings using a more physiological culture system (46), we recreated, at least partially, the pathological conditions of an inflamed synovium by culturing RA-FLS in media supplemented with 30% SF obtained from arthritis patients. Rather than using a chemically defined medium and only one stimulus, such as a single recombinant cytokine, this *ex vivo* model provides a means to activate FLS with the mixture of pro-inflammatory and anti-inflammatory mediators that are present in the SF of diseased joints. We found that the addition of SF to RA-FLS significantly increased the production of IL-6. This effect is mediated almost completely by the proNGF present in SF, as the inhibition of proNGF binding to p75NTR led to an 80% reduction in IL-6 production. As it is well known that IL-1β enhances the expression levels of various inflammatory factors in RA-FLS through the activation of NF-kB/ERK/STAT1 axis, p38 mitogen-activated protein kinase (MAPK) and JUNK signaling pathways (22, 32, 33, 47), we compared the effects of IL-1β and p75NTR neutralization on inflammatory cytokine production. We found that in RA-FLS the inhibition of p75NTR activation by LM11A-31 reduces the release of IL-6 to a similar extent as IL-1β inhibition by canakinumab. Although these data are limited to an *ex vivo* culture system, they nonetheless suggest that inhibition of p75NTR is, at least, as effective as the inhibition of a potent activator of pro-inflammatory response in RA-FLS as IL-1β.

In neurons, proNGF binding to p75NTR activates intracellular pathways that involve JNK and lead to cell death (48–50). Our results demonstrated that the effects of p75NTR activation or inhibition were not due to changes in cell viability of RA-FLS.

We also provide an initial characterization of the intracellular pathway activated by p75NTR in RA-FLS. We investigated the JNK and p38 MAPK-induced pathways that regulate pro-inflammatory cytokines and metalloproteinase expression, matrix degradation and joint destruction (34, 51). Activated RA-FLS are characterized by p38 and JNK phosphorylation. Inhibition of p75NTR by LM11A-31 effectively reduces phosphorylation of both MAPKs, demonstrating their involvement in p75NTR signal transduction in RA-FLS. This observation is consistent with the known role of these MAPKs in mediating pro-inflammatory responses (48).

Preliminary data in other inflammatory conditions in mice indicate that the involvement of p75NTR in regulating inflammatory pathways may not be restricted to arthritis. In murine streptozotocin-induced diabetes, accumulation of proNGF and enhanced p75NTR expression are present in the retina. p75NTR-KO mice are protected from diabetes-induced retinal inflammation and show decreased TNF-α expression (52). Treatment of streptozotocin-treated mice with LM11A-31 significantly inhibits an increase in serum TNF-α and IL-1β (53). Similarly, in sepsis-induced neuroinflammation in mouse hippocampus, LM11A-31 treatment causes a decrease in IL-1β concentrations, associated with a reduction in JNK phosphorylation (53).

In conclusion, we demonstrate that *in vivo*, in the synovium of treatment-naïve RA patients, the clusters of FLS typically involved in joint inflammation and damage show a distinctive overexpression of p75NTR. Complementing this finding, we demonstrated *in vitro* that RA-FLS are characterized by a high expression of p75NTR and by a high production of proNGF that, together, promote an autocrine loop that enhances RA-FLS inflammatory responses. Altogether, our results suggest that an active proNGF/p75NTR axis contributes to the chronicity of synovial inflammation and that the proNGF/p75NTR pathway could represent a novel target in the treatment of chronic arthritis.

New approaches in arthritis based on novel mechanisms of actions and targets are needed since many patients do not reach remission and continue to accumulate damage, with significant social and economic costs. Thus, the demonstration of the novel pro-inflammatory role of p75NTR and its preferential ligand, proNGF, in the amplification of inflammatory responses and the preclinical studies with specific receptor inhibitors together will provide the rationale for targeting p75NTR in chronic arthritis and potentially in other chronic inflammatory diseases.

At present the safety of p75NTR inhibition with LM11A-31 is being investigated in a phase 2 trial (ClinicalTrials.gov Identifier: NCT03069014) to test its use in the treatment of Alzheimer’s disease patients.

## Data Availability Statement

The original contributions presented in the study are publicly available. This data can be found here: www.ebi.ac.uk/arrayexpress/experiments/E-MTAB-8322/.

## Ethics Statement

The studies involving human participants were reviewed and approved by Fondazione Policlinico Universitario A. Gemelli IRCCS, Institutional Ethics Committee (ID#6334/15) Bambino Gesú Children’s Hospital IRCCS ethical committee (ID#2333_OPBG_2020). Written informed consent to participate in this study was provided by the participants’ legal guardian/next of kin.

## Author Contributions

LF, GM, IC, LMD,MS, EZ, BT, and LC contributed to acquisition of data. LB-L, SA, BT, LM, EG, MK-S, AA, SM-M, and FDB contributed to analysis and interpretation of data. LB-L, LF, FDB, LM, MS, EG, SC, and SA drafted the article and revised it critically. All authors contributed to the article and approved the submitted version.

## Funding

This research was supported by the Ministry for Economic Development (Call for the implementation of patent enhancement programs through the funding of Proof of Concept (PoC) projects of Italian Universities and Bodies Italian Public Research (EPR) Institutes and Scientific Institutes for Treatment and Inpatient Care (IRCCS)- Enhancement program “ELEVATOR-mEdicaL patEnts VAlorizaTiOn pRogram”), by Ricerca Corrente grants from the Italian Ministry of Health to FDB and LB-L and by Fondazione Bambino Gesu’grant to LB-L. Single-cell RNA sequencing data were funded by Ricerca Finalizzata Ministero della Salute (GR-2018-12366992) for SA and Research into Inflammatory Arthritis Centre Versus Arthritis (RACE 20298 & 22072) for MK-S. The grantors were not involved in the study design, or in the analysis and interpretation of the data.

## Conflict of Interest

FDB has received grants from Abbvie, Novimmune, Sobi, Novartis, Roche, Sanofi, all unconnected with the submitted work. LB-L and FDB are the inventors and OPBG and CNR are the owners of an issued European Patent EP2667895.

The remaining authors declare that the research was conducted in the absence of any commercial or financial relationships that could be construed as a potential conflict of interest.

## Publisher’s Note

All claims expressed in this article are solely those of the authors and do not necessarily represent those of their affiliated organizations, or those of the publisher, the editors and the reviewers. Any product that may be evaluated in this article, or claim that may be made by its manufacturer, is not guaranteed or endorsed by the publisher.

## Supplementary Material

Supplementary Figure 1 Violin plots showing NGF, NGFR, NTRK1 and SORT1 gene expression in distinct stromal cells clusters from synovial tissue of naive to treatment RA (n=4) and RA in sustained clinical and ultrasound remission (n=4). Data are reported as mean (white dot).

Supplementary Figure 2
**(A)** Western Blot for p75NTR confirmed protein overexpression of p75NTR in RA-FLS (n=3) compared to skin-FB (CTRL). **(B)** Sortilin expression in RA-FLS (n=8), OA-FLS (n=6) and CTRL-FB (n=5) showed the same expression pattern of p75NTR. **(C)** Increase of p75NTR expression in RA-FLS is dose–dependently induced by IL-1β. Results are compared to unstimulated condition and calculated as Fold Change (F.C.) and represent the mean ± SEM of 5 independent experiments. Differences between groups were analyzed using unpaired *t*-test (*p<0.05, **p<0.01). **(D)** RA-FLS stimulated with different doses of IL-1β showed a dose-dependent increase of NGF mRNA. The data represent the mean ± SEM of 5 independent experiments. Results were compared to unstimulated condition and calculated as Fold Change (F.C.) using unpaired *t*-test (**p<0.01, ***p<0.001).

Supplementary Figure 3 LM11A-31 (10nM) was used to inhibit proNGF binding to p75NTR in RA-FLS activated using 100 ng/ml TNF-α, 100 ng/ml LPS or 1 ng/ml IL-1β. The production of IL-6 **(A)**, IL-8 **(B)** and MCP1 **(C)** in RA-FLS cultured in 10% FBS DMEM was measured in the conditioned media after 18 hours of incubation. p75NTR was neutralized using a specific anti-p75NTR antibody (2,5 µg/ml) and the release of IL-6 was measured in RA-FLS activated with 100 ng/ml TNF-α **(D)**, 100 ng/ml LPS **(E)** or 1ng/ml IL-1β **(F)**. In this set of experiments (n=6) cells were cultured in 10% FBS DMEM for 18 hours. All the results are expressed as pg/ml.

Supplementary Figure 4
**(A)** The addition of exogenous proNGF (200ng/ml) to RA-FLS, cultured in a chemically defined medium, enhances the production of IL-6 in RA-FLS activated with sub-optimal doses of IL-1β (IL-1β 10-50-100 pg/ml). The increase of IL-6 induced by exogenous proNGF (IL-1b+pNGF) is blocked by p75NTR inhibition with 10nM LM11A-31 (IL-1b+i31). IL-1β-activated RA-FLS show a similar IL-6 reduction when p75NTR was inhibited (IL-1b+i31). To interpret this data, it should be considered that LM11A-31 prevents the binding of the endogenously-produced proNGF to p75NTR, whose synthesis is strongly activated in RA-FLS by IL-1β stimulation (see **Figures 3A**–**C**, **Supplementary 2D**). The data represent the mean ± SEM, results were analyzed using paired *t*-test (*p<0.05). **(B)** Western Blot analysis showed that almost only proNGF forms were detectable in synovial fluids of patients (40 μg total protein). Very low protein levels of mature NGF were observed. 10% reducing gels were incubated with anti-NGF M20 antibody that recognizes both mNGF and proNGF forms.
