# Supplementary figures and images for: Pro Nerve Growth Factor and Its Receptor p75NTR Activate Inflammatory Responses in Synovial Fibroblasts: A Novel Targetable Mechanism in Arthritis
Source: Front Immunol. 2022 Mar 4;13:818630. doi: 10.3389/fimmu.2022.818630 (PMC8931659; doi:10.3389/fimmu.2022.818630)

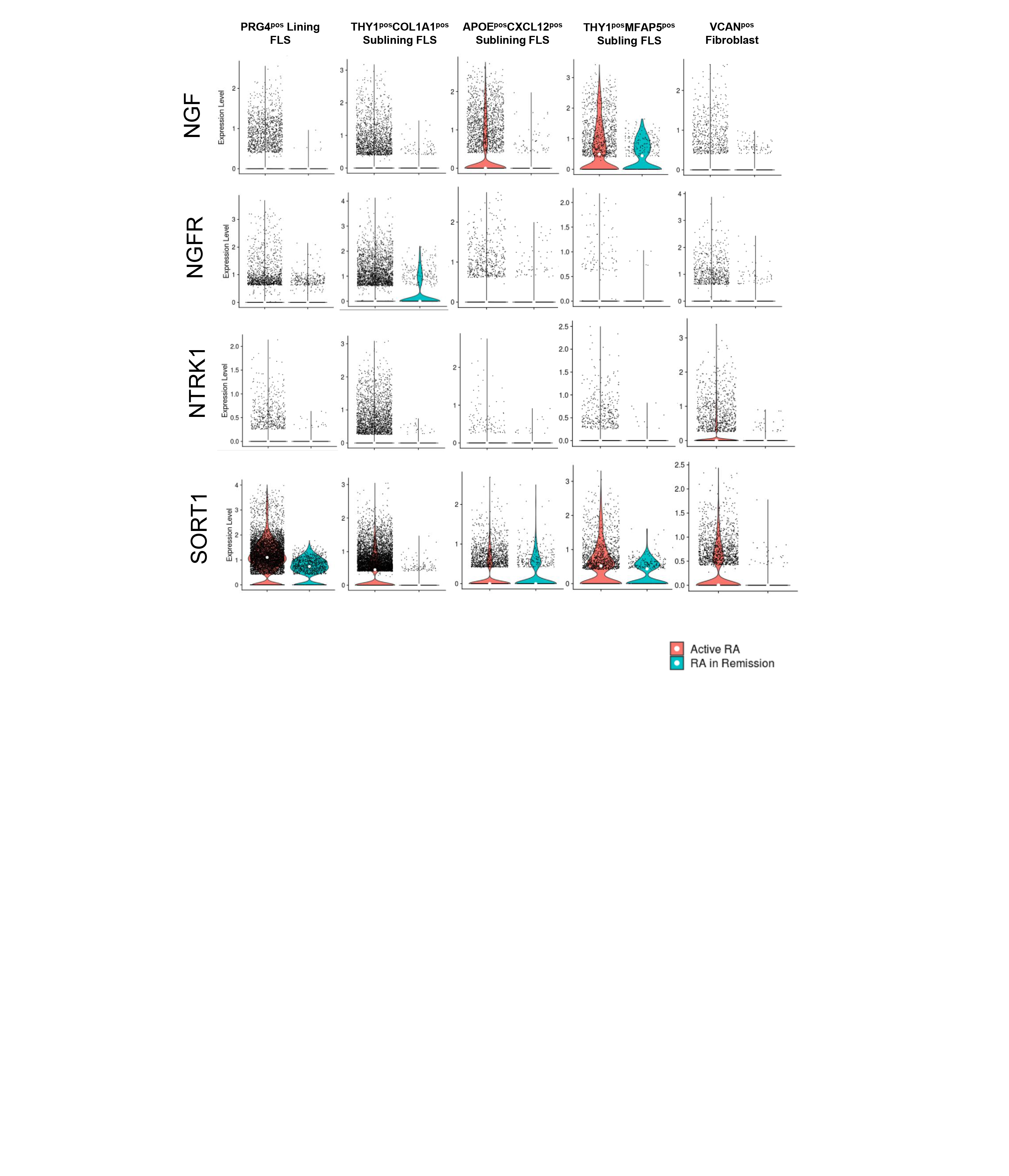

Supplement: Supplementary Figure 1 — Violin plots showing NGF, NGFR, NTRK1 and SORT1 gene expression in distinct stromal cells clusters from synovial tissue of naive to treatment RA (n=4) and RA in sustained clinical and ultrasound remission (n=4). Data are reported as mean (white dot). [file Image_1.jpeg]

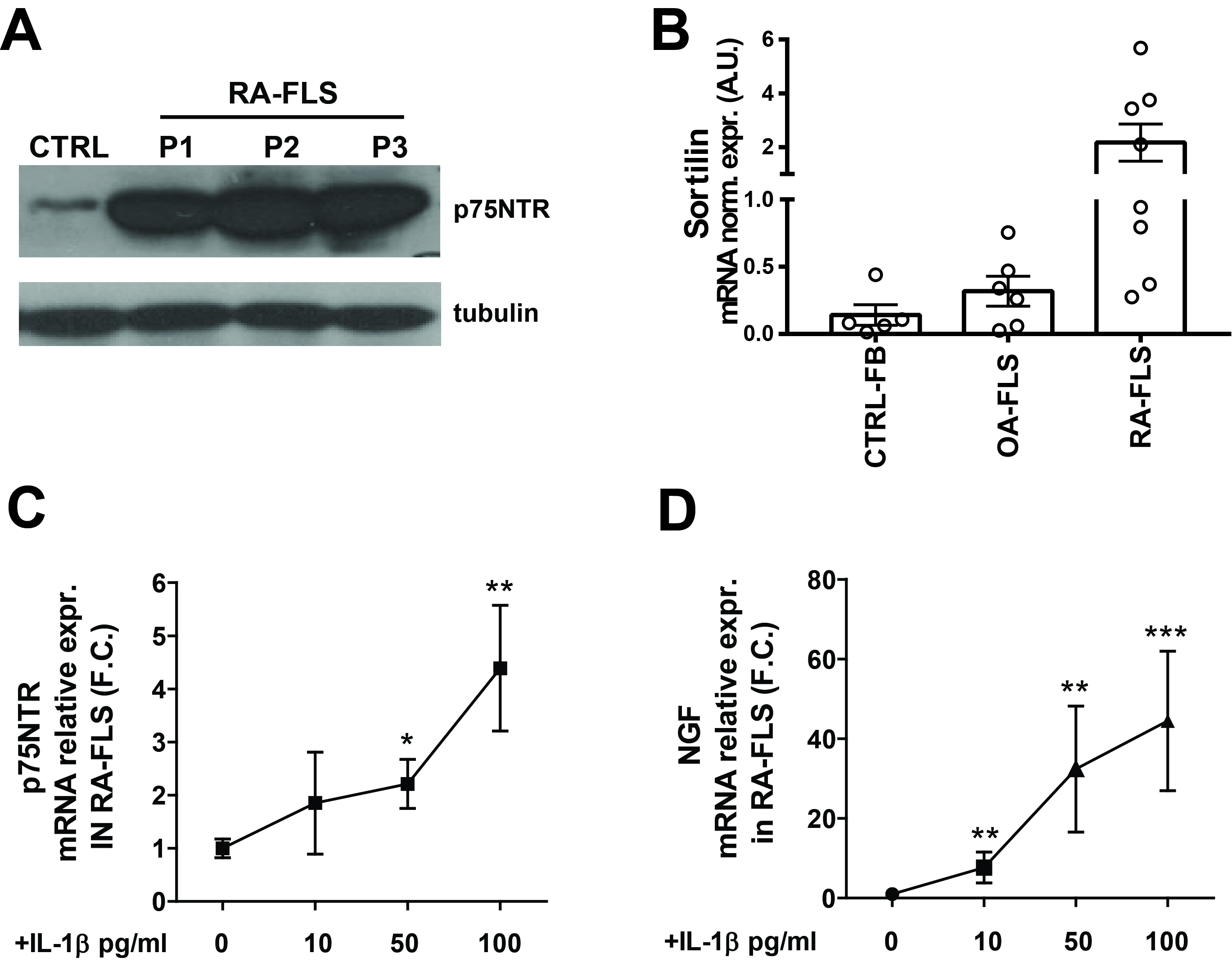

Supplement: Supplementary Figure 2 — (A) Western Blot for p75NTR confirmed protein overexpression of p75NTR in RA-FLS (n=3) compared to skin-FB (CTRL). (B) Sortilin expression in RA-FLS (n=8), OA-FLS (n=6) and CTRL-FB (n=5) showed the same expression pattern of p75NTR. (C) Increase of p75NTR expression in RA-FLS is dose–dependently induced by IL-1β. Results are compared to unstimulated condition and calculated as Fold Change (F.C.) and represent the mean ± SEM of 5 independent experiments. Differences between groups were analyzed using unpaired t-test (*p<0.05, **p<0.01). (D) RA-FLS stimulated with different doses of IL-1β showed a dose-dependent increase of NGF mRNA. The data represent the mean ± SEM of 5 independent experiments. Results were compared to unstimulated condition and calculated as Fold Change (F.C.) using unpaired t-test (**p<0.01, ***p<0.001). [file Image_2.jpeg]

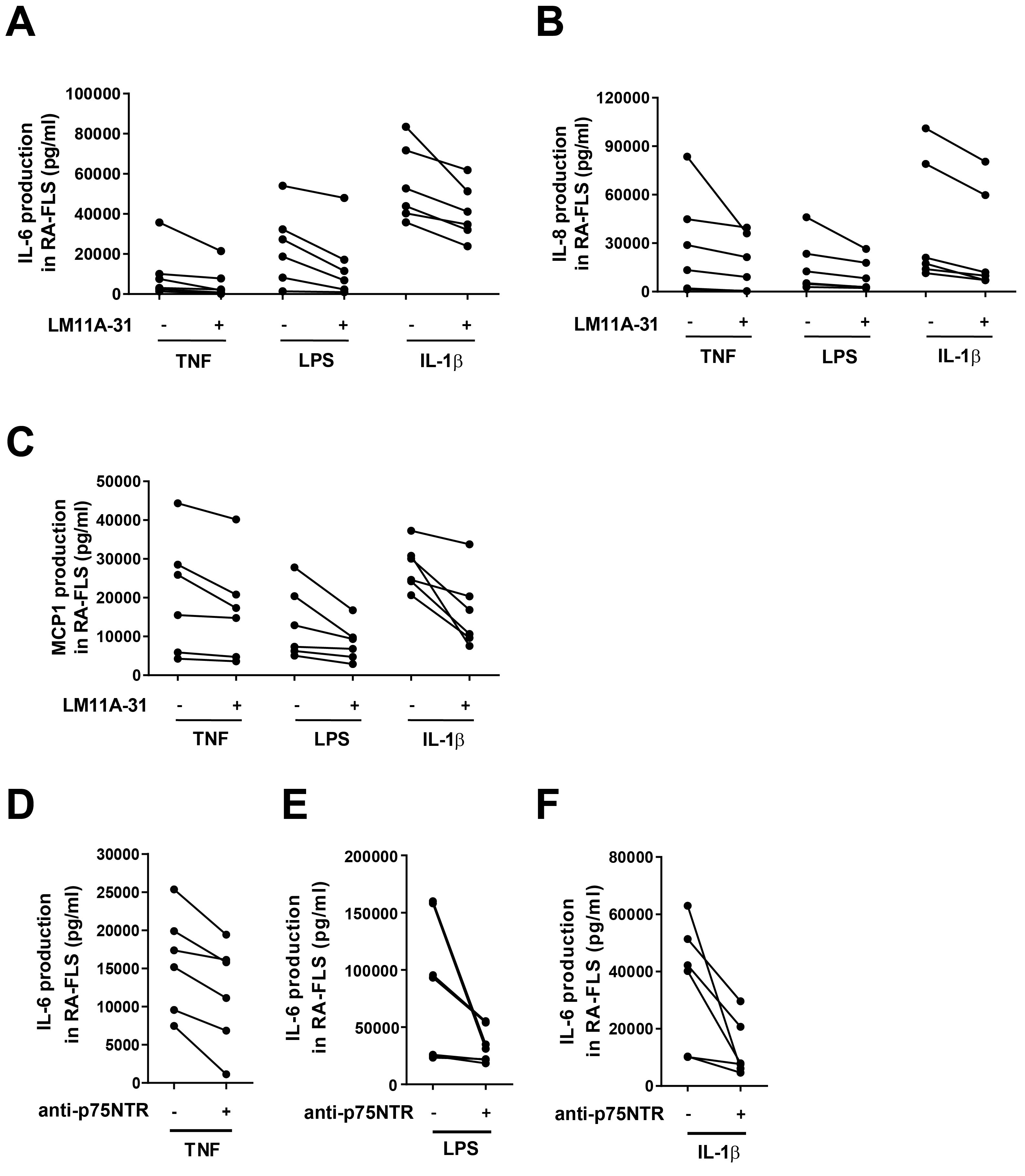

Supplement: Supplementary Figure 3 — LM11A-31 (10nM) was used to inhibit proNGF binding to p75NTR in RA-FLS activated using 100 ng/ml TNF-α, 100 ng/ml LPS or 1 ng/ml IL-1β. The production of IL-6 (A), IL-8 (B) and MCP1 (C) in RA-FLS cultured in 10% FBS DMEM was measured in the conditioned media after 18 hours of incubation. p75NTR was neutralized using a specific anti-p75NTR antibody (2,5 µg/ml) and the release of IL-6 was measured in RA-FLS activated with 100 ng/ml TNF-α (D), 100 ng/ml LPS (E) or 1ng/ml IL-1β (F). In this set of experiments (n=6) cells were cultured in 10% FBS DMEM for 18 hours. All the results are expressed as pg/ml. [file Image_3.jpeg]

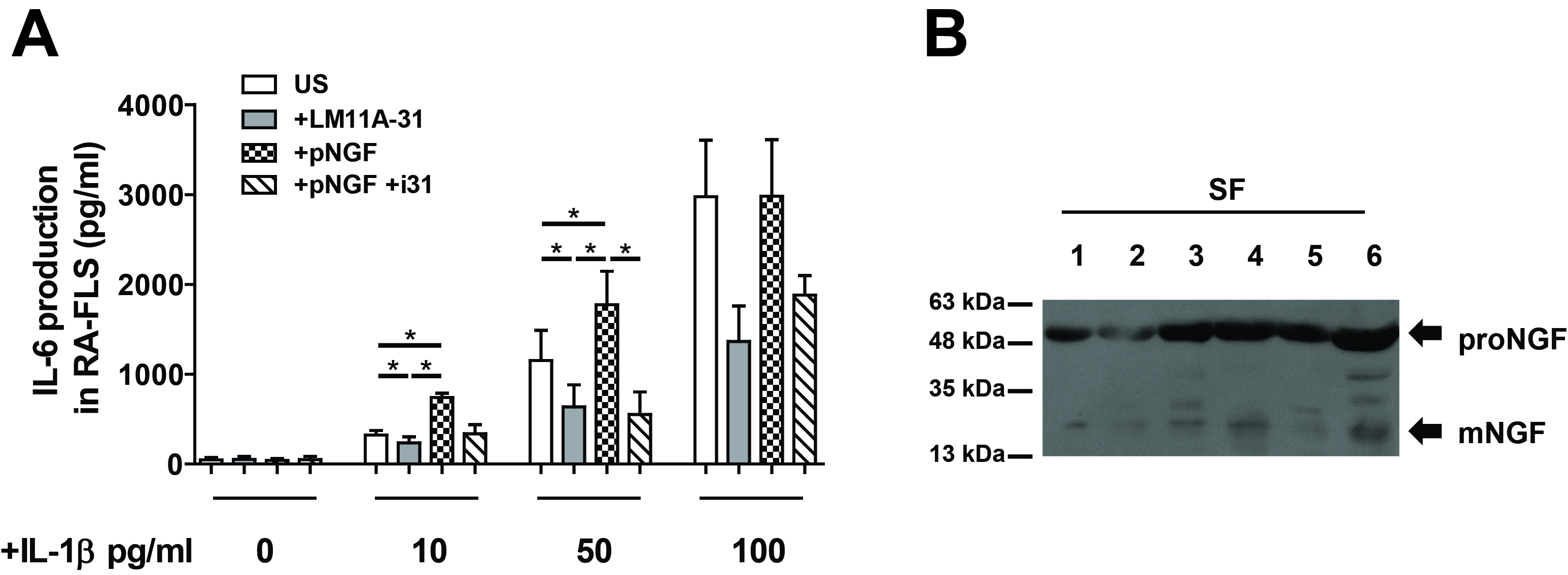

Supplement: Supplementary Figure 4 — (A) The addition of exogenous proNGF (200ng/ml) to RA-FLS, cultured in a chemically defined medium, enhances the production of IL-6 in RA-FLS activated with sub-optimal doses of IL-1β (IL-1β 10-50-100 pg/ml). The increase of IL-6 induced by exogenous proNGF (IL-1b+pNGF) is blocked by p75NTR inhibition with 10nM LM11A-31 (IL-1b+i31). IL-1β-activated RA-FLS show a similar IL-6 reduction when p75NTR was inhibited (IL-1b+i31). To interpret this data, it should be considered that LM11A-31 prevents the binding of the endogenously-produced proNGF to p75NTR, whose synthesis is strongly activated in RA-FLS by IL-1β stimulation (see Figures 3A – C , Supplementary 2D ). The data represent the mean ± SEM, results were analyzed using paired t-test (*p<0.05). (B) Western Blot analysis showed that almost only proNGF forms were detectable in synovial fluids of patients (40 μg total protein). Very low protein levels of mature NGF were observed. 10% reducing gels were incubated with anti-NGF M20 antibody that recognizes both mNGF and proNGF forms. [file Image_4.jpeg]
